# Proof-of-concept trial results of the HeartMan mobile personal health system for self-management in congestive heart failure

**DOI:** 10.1038/s41598-021-84920-4

**Published:** 2021-03-11

**Authors:** Els Clays, Paolo Emilio Puddu, Mitja Luštrek, Giovanni Pioggia, Jan Derboven, Marilena Vrana, Johan De Sutter, Rita Le Donne, Anneleen Baert, Marko Bohanec, Maria Costanza Ciancarelli, Amos Adeyemo Dawodu, Michel De Pauw, Delphine De Smedt, Flavia Marino, Sofie Pardaens, Michele Salvatore Schiariti, Jakob Valič, Marc Vanderheyden, Aljoša Vodopija, Gennaro Tartarisco

**Affiliations:** 1 Department of Public Health and Primary Care, Ghent University, Campus University Hospital Ghent, entrance 42, 4K3, Corneel Heymanslaan 10, 9000 Ghent, Belgium; 2 Department of Cardiovascular, Respiratory, Nephrological, Anesthesiological and Geriatric Sciences, Sapienza University of Rome, Rome, Italy; 3 Department of Intelligent Systems, Jožef Stefan Institute, Ljubljana, Slovenia; 4 Institute for Biomedical Research and Innovation, Italian National Research Council, Messina, Italy; 5 Meaningful Interactions Lab, KU Leuven, Leuven, Belgium; 6 European Heart Network, Brussels, Belgium; 7 Department of Cardiology, AZ Maria Middelares Ghent, Ghent, Belgium; 8 Department of Internal Medicine, Ghent University, Ghent, Belgium; 9 Complex Operative Unit of Pneumology and Telemedicine, San Camillo de Lellis General Hospital, Rieti, Italy; 10 Department of Knowledge Technologies, Jožef Stefan Institute, Ljubljana, Slovenia; 11 Cardiovascular Center, Onze-Lieve-Vrouw Hospital Aalst, Aalst, Belgium

**Keywords:** Quality of life, Disease prevention, Heart failure

## Abstract

This study tested the effectiveness of HeartMan—a mobile personal health system offering decisional support for management of congestive heart failure (CHF)—on health-related quality of life (HRQoL), self-management, exercise capacity, illness perception, mental and sexual health. A randomized controlled proof-of-concept trial (1:2 ratio of control:intervention) was set up with ambulatory CHF patients in stable condition in Belgium and Italy. Data were collected by means of a 6-min walking test and a number of standardized questionnaire instruments. A total of 56 (34 intervention and 22 control group) participants completed the study (77% male; mean age 63 years, sd 10.5). All depression and anxiety dimensions decreased in the intervention group (*p* < 0.001), while the need for sexual counselling decreased in the control group (*p* < 0.05). Although the group differences were not significant, self-care increased (*p* < 0.05), and sexual problems decreased (*p* < 0.05) in the intervention group only. No significant intervention effects were observed for HRQoL, self-care confidence, illness perception and exercise capacity. Overall, results of this proof-of-concept trial suggest that the HeartMan personal health system significantly improved mental and sexual health and self-care behaviour in CHF patients. These observations were in contrast to the lack of intervention effects on HRQoL, illness perception and exercise capacity.

## Introduction

Congestive heart failure (CHF) is a common cardiovascular disease, with an estimated 26 million adults worldwide living with this condition and its prevalence rising to ≥ 10% among people over 70 years of age^1,2^. Despite major improvements in medical treatment, CHF remains a condition with high rates of premature mortality and hospital readmission^3^. Since there is no cure available, proper disease management in CHF is crucial as it may relieve symptoms, prevent hospitalisation or decrease mortality^2^. Optimal management of the disease may also affect the patient’s health-related quality of life (HRQoL), considered to be an essential treatment goal^4^. Despite the availability of evidence-based guidelines for proper disease management, the uptake of these guidelines in clinical practice is generally poor, especially when it comes to meeting the recommendations for physical exercise^5^. Self-care in CHF management is highly complex since extensive behavioural efforts are required relating to medication adherence, fluid and sodium intake, healthy diet and weight management, smoking and alcohol consumption, and physical exercise^6^. Patients with CHF—who are typically older persons and frequently suffer from comorbidities and mental health impairment—often struggle to adhere to their complicated treatment scheme and lifestyle advice to manage their disease. Therefore, the use of mHealth applications is promising and holds large potential to optimize self-care and improve clinical outcomes. Results on the effectiveness of mHealth interventions in CHF are however inconclusive^7–10^.

HeartMan was developed as a comprehensive personal health system to address CHF self-management^11^. The core of the system is a mobile application, which is connected to a number of sensing devices—including a custom wristband—and cloud services. A decision support system (DSS) provides recommendations that are shown in the mobile application, which also collects inputs from the patients. It is a multi-disciplinary system combining a number of different intervention modalities to cover both physical health management and psychological support. The expert system for physical health in the DSS includes a comprehensive exercise program with an individualized weekly plan consisting of endurance and resistance exercises. It further comprises personalized systems for nutrition advice, self-monitoring, medication intake and disease education. The expert system for psychological support provides cognitive behavioral interventions and mindfulness exercises implemented according to a weekly plan. Unlike most mHealth interventions targeting hard clinical endpoints like mortality and hospitalisation, the primary outcomes of HeartMan are HRQoL and self-management. The therapeutic goal in CHF is not only prolonging survival, but also improving the quality of life gained. Hard endpoints do not necessarily reflect how a disease, its symptoms and treatment are experienced by the patient. Consequently, an increasing interest occurred in using patient-reported outcomes (PROs) as independent outcome measures in clinical practice over the past years^12^. Moreover, it has been demonstrated that PROs like self-reported HRQoL and health status have a pathophysiological basis and are predictors of clinical events in patients with CHF^13,14^.

The aim was to evaluate the effectiveness of HeartMan, within a randomized controlled proof-of-concept trial, on HRQoL and self-management as primary endpoints, and on exercise capacity, illness perception, and mental and sexual health as the secondary endpoints.

## Methods

### Study design and population

The HeartMan randomized controlled proof-of-concept trial was implemented in 3 hospitals in Belgium (recruitment from 04/01/2018 until 27/06/2018) and one hospital and a local health authority in Italy (recruitment from 11/07/2018 until 06/03/2019), details have been described elsewhere^11^. The design used a 1:2 randomization ratio of control group receiving standard care vs. intervention group receiving the HeartMan system on top of standard care. The main inclusion criteria were being an adult ambulatory CHF patient (both ischemic or non-ischemic aetiology) in stable condition, having a Functional New York Heart Association (NYHA) class of 2 or 3, and having a reduced left ventricular ejection fraction (LVEF) of ≤ 40%.

**Figure 1:**
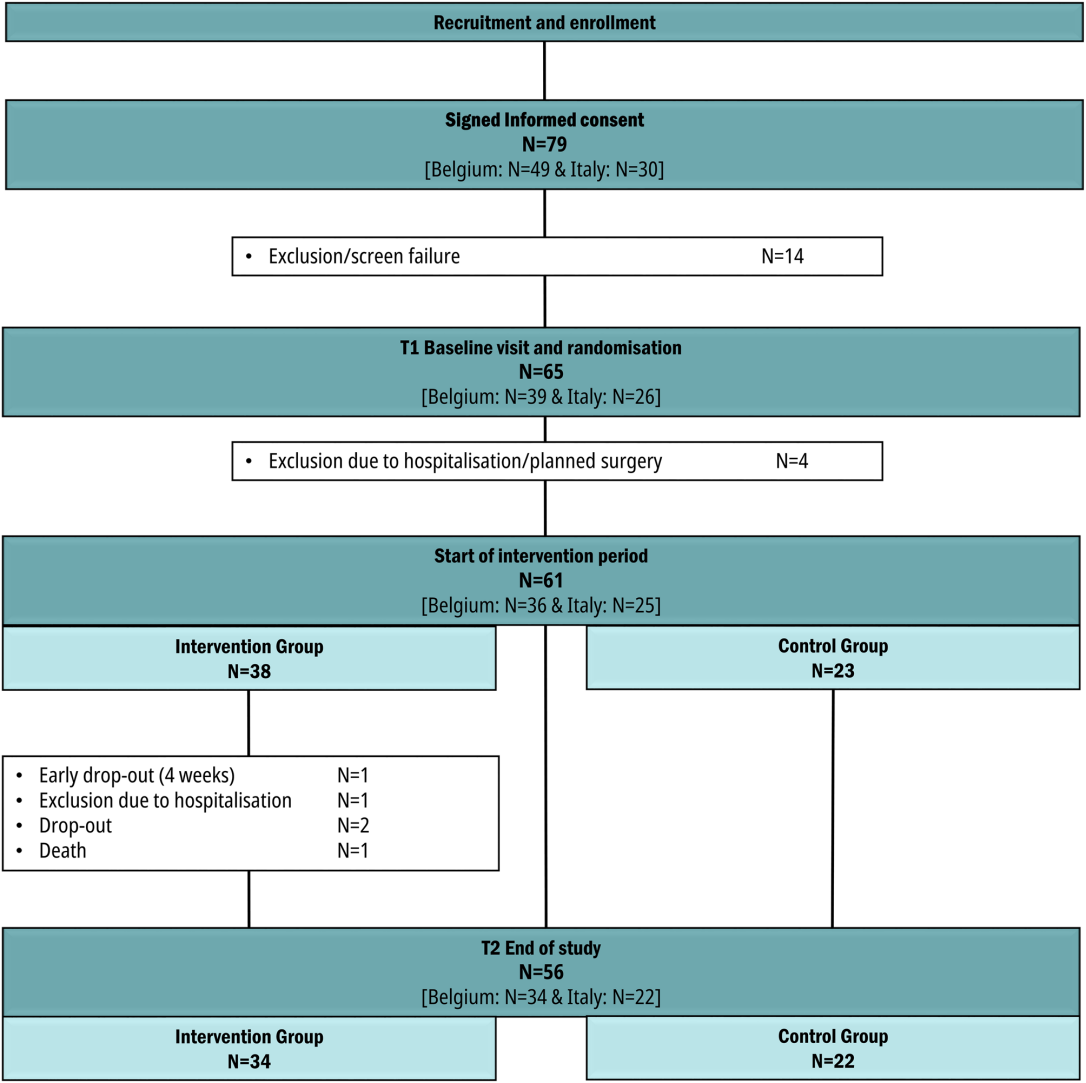
Participant flowchart.

An a-priori sample size of 120 participants (80 intervention and 40 control patients) was targeted, based on power calculations that considered heart rate changes—a parameter correlating with HRQoL—as derived from the CHIRON study which was undertaken by part of the current investigators group^14,15^. It was anticipated that 90 patients were needed to show an 5.8 beats per minute difference in average daily awake heart rate with 90% power between the two groups. This targeted number was increased to 120 (60 in each participating country) to account for drop-out. After recruitment by the treating cardiologist or general physician, 79 patients signed informed consent, 14 of whom were excluded because they did not fulfil the inclusion criteria or because their health deteriorated shortly after. Of the 65 patients being randomized, 4 patients were excluded due to hospitalisation before the start of the intervention, and 5 were excluded during the intervention period due to withdrawal or clinical event, leaving a sample of 56 participants—i.e. 34 in the intervention and 22 in the control group—to evaluate the intervention effects (see Fig. 1 for the participant flowchart).

This study complies with the Declaration of Helsinki and in both countries, formal approval was obtained from the ethical committees (Central Ethical Committee of the University Hospital Ghent in Ghent, Belgium and the Central Ethical Committee of Lazio 1 of San Camillo-Forlanini Hospital in Rome, Italy) and from the national institutions for clinical trials (the Belgian Federal Agency for Medicines and Health Products and the Ministry of Health in Italy). The study was registered at ClinicalTrials.gov on 13/04/2018 (NCT03497871).

### Procedure and intervention

Electronic health records of the target population were screened for eligibility. At the time of a regular outpatient visit, information about the study was presented to eligible patients by the treating physician or heart failure nurse, after which interested patients signed informed consent. The participants returned to the hospital for a subsequent appointment, during which baseline data were collected by means of clinical and questionnaire assessments. Next, the patients were randomly assigned to either the control group or the intervention group with a 1:2 ratio, using a system with two balanced series (one in each country) of sealed envelopes. All outcome measurements were repeated in both intervention and control group at the end of the trial.

Patients in the control group received usual care, i.e. the standard treatment in line with clinical guidelines offered by the cardiologist, general practitioner and CHF nurse. Patients in the intervention group continued to receive usual care, and additionally used the HeartMan personal health system in their home setting. The intervention was initiated during a home visit by a member of the research team, providing all necessary equipment, technical installation and user instructions.

The trial equipment included an off-the-shelf blood pressure monitor (A&D Medical, Model Number UA-611), weight scale (ADE, Model Silje BE1303) and pill organizer (PuTwo, 7-Day AM/PM Night Reminder Medi-Planner), and a custom wristband sensor developed by BITTIUM (Oulo, Finland) recording heart rate (variability), galvanic skin response, skin temperature and acceleration. Based on data collected from the sensing devices and patient monitoring methods, the DSS recommends actions that are presented to the patient via a mobile application on a smartphone (Nokia 6 TA_1021) which was provided for the duration of the trial. The main components of the DSS were an expert system to manage physical health (including personalized physical exercise scheme, lifestyle advice on nutrition, notification system for medication intake and self-monitoring) and an expert system providing psychological support (including elements of cognitive behavioural therapy and mindfulness exercises), in addition to the availability of overall disease education about CHF and its treatment. Intervention patients used the HeartMan system for a period of 3 up to 6 months, during which a telephone-based helpdesk was operational for addressing technical difficulties and user problems.

### Data collection

The outcome measures were collected at baseline (T1) and at the end-of-study visit (T2) in the hospital setting. In line with the recommended standardized protocol of the European Heart Failure Association, a 6-min walking test (6MWT) was conducted by a trained researcher^5^. The number of meters walked within 6 min was registered to evaluate exercise capacity. Resting heart rate was measured before the test (using a UA-611 monitor, A&D Medical) and used as additional clinical outcome measure.

The patients filled in a number of standardized questionnaire instruments during the hospital visit. Completion of the self-administered questionnaire package took on average 30 min and was done in the presence of a member from the research team (in case support or clarification was needed). The Self-Care of Heart Failure Index (SCHFI) was used to measure self-care maintenance (10 items) and self-care confidence (6 items), with each subscale being standardized to a score ranging from 0 to 100, higher scores indicating better self-care quality^16^. The 21-item disease-specific Minnesota Living with Heart Failure Questionnaire (MLHFQ) was used to assess overall HRQoL, with a higher score indicating a more impaired HRQoL, as well as the sub-scales for impaired physical HRQoL and impaired emotional HRQoL, calculated from a subset of 8 and 5 items, respectively^17^. The brief Illness Perception Questionnaire (IPQ) provided a score calculated from 8 items, with a higher score representing a higher degree to which the illness is perceived as threatening or benign, on a scale from 0 to 10^18^. The 21-item Beck Depression Inventory II (BDI II) instrument returned a total score and two sub-scores on somatic-affective and cognitive manifestations of depression^19^. We used the 40-item State Trait Anxiety Inventory Form Y (STAI-Y) to assess *state* anxiety (Y1: how the subject feels at the time of questionnaire submission) and *trait* anxiety (Y2: how the subject feels normally)^20^. The Sexual Adjustment Scale (SAS) consists of six items and yields a score varying from 0 to 18, with a higher score indicating more sexual disturbance experienced^21^. The Needs for Counseling scale in CHF (NSCS-CHF) includes 18 items from which a score from 1 (totally unimportant) to 4 (very important) is calculated, with a higher score indicating a higher expressed need for counseling about sexual activity in CHF^22^.

Electronic health records were screened to obtain data on clinical characteristics (body mass index, laboratory parameters), CHF-related characteristics (NYHA class, aetiology, date of first episode, LVEF measured by 2D echocardiography), and information about comorbidities and medication use. In case the necessary electronic health data were available, the predicted one-year mortality risk was calculated using the MAGGIC^23^ and 3C-HF^24^ scoring systems. If a new assessment of LVEF was available at the end of the intervention study, this was also registered as an additional clinical outcome measure in a sub-sample of participants.

### Statistical analysis

Continuous variables were checked for normality of distribution before analysis. Descriptive statistics were obtained through proportions/numbers and mean (plus standard deviation (sd)) or median (plus interquartile range (IQR)) values. Descriptive, clinical and outcome variables at T1 were compared between intervention and control group by means of independent samples Student’s t-test, Mann–Whitney *U* test, Chi² or Fisher’s Exact test. The same comparative statistical tests were used for the drop-out analysis and for evaluating potential differences at T1 between Italian and Belgian participants. To evaluate the intervention effects, we assessed the change in the outcome variables from T1 to T2 in the intervention and control group by means of the paired-samples Student’s t-test or Wilcoxon signed-rank test. Group differences in these change scores were furthermore evaluated with independent samples Student’s t-test or Mann–Whitney *U* test. A *p*-value at < 0.05 level was considered statistically significant. All analyses were conducted in IBM SPSS Statistics version 24.

## Results

The sample of 56 patients had a mean age of 63 years (sd 10.5), was predominantly male (77%), and had by design a reduced LVEF of ≤ 40% (mean 32%; sd 6.3). An overview of characteristics and outcome variables at T1 is shown in Table 1. Demographic, clinical and other outcome variables were well balanced between the intervention and control group.

**Table 1:** Descriptive characteristics of socio-demographic, clinical and outcome variables at T1 in total sample, intervention and control group.

Baseline characteristics and outcome variables	N missing	Total sample (N = 56)	Intervention group (N = 34)	Control group (N = 22)	*p-value* group comparison
Age (years): mean (sd)	0	63.1 (10.5)	61.8 (11.0)	65.2 (9.6)	0.23^a^
Aged ≥ 60 years: N (%)	0	34 (60.7)	20 (58.8)	14 (63.6)	0.72^b^
Female sex: N (%)	0	13 (23.2)	8 (23.5)	5 (22.7)	0.95^b^
Country: N (%)	0				0.84^b^
Belgium		34 (60.7)	21 (61.8)	13 (59.1)	
Italy		22 (39.3)	13 (38.2)	9 (40.9)	
Body Mass Index (kg/m²): mean (sd)	0	29.0 (5.3)	29.5 (5.4)	28.2 (5.2)	0.36^a^
Weight group: N (%)	0				0.35^b^
Normal weight (BMI < 25 kg/m²)		9 (16.1)	6 (17.6)	3 (13.6)	
Overweight (25 kg/m² ≤ BMI < 30 kg/m²)		29 (51.8)	15 (44.1)	14 (63.6)	
Obese (BMI ≥ 30 kg/m²)		18 (32.1)	13 (38.2)	5 (22.7)	
NYHA class: N (%)	3				0.69^b^
NYHA class II		46 (86.8)	26 (83.9)	20 (90.9)	
NYHA class III		7 (13.2)	5 (16.1)	2 (9.1)	
Ischemic CHF aetiology: N (%)	3	30 (56.6)	19 (55.9)	11 (57.9)	0.89^b^
First episode CHF < 18 months: N (%)	3	9 (17.0)	7 (20.6)	2 (10.5)	0.46^c^
LVEF (%): mean (sd)	0	32.2 (6.3)	32.7 (5.9)	31.3 (6.9)	0.41^a^
MAGGIC (% 1-year mortality risk): median (IQR)	7	14.7 (9.8;32.9)	14.6 (9.2;29.2)	16.0 (12.2;35.6)	0.31^d^
3C-HF (% 1-year mortality risk): median (IQR)	5	6.0 (2.0;12.0)	5.0 (1.0;11.3)	6.0 (2.0;16.5)	0.54^d^
Comorbidities: N (%)					
Hypertension	2	30 (55.6)	18 (56.3)	12 (54.5)	0.90^b^
Hyperlipidaemia	2	40 (74.1)	22 (66.7)	18 (85.7)	0.12^b^
Diabetes	1	23 (41.8)	16 (47.1)	7 (33.3)	0.32^b^
COPD	7	7 (14.3)	4 (12.9)	3 (16.7)	0.70^c^
CVA/TIA	0	12 (21.4)	5 (14.7)	7 (31.8)	0.18^c^
Peripheral vessel disease	1	7 (12.7)	4 (12.1)	3 (13.6)	1.00^c^
Chronic kidney dysfunction	2	3 (5.6)	2 (6.3)	1 (4.5)	1.00^c^
Anaemia	2	2 (3.7)	1 (3.0)	1 (4.8)	1.00^c^
Self-care maintenance (SCHFI) (0–100): mean (sd)	0	59.7 (15.7)	61.4 (16.8)	57.1 (13.8)	0.33^a^
Self-care confidence (SCHFI) (0–100): mean (sd)	4	62.5 (21.3)	65.1 (20.8)	58.8 (21.9)	0.30^a^
Impaired HRQoL total (MLHFQ) (0–105): mean (sd)	0	31.3 (19.6)	32.1 (22.9)	30.0 (13.5)	0.66^a^
Impaired HRQoL physical (MLHFQ) (0–40): mean (sd)	0	14.3 (10.3)	14.7 (11.9)	13.7 (7.6)	0.72^a^
Impaired HRQoL emotional (MLHFQ) (0–25): mean (sd)	0	7.1 (5.9)	7.5 (6.6)	6.4 (4.7)	0.46^a^
Distance 6MWT (meters): mean (sd)	3	396.0 (124.1)	384.4 (127.3)	415.3 (119.3)	0.38^a^
Resting heart rate before 6MWT (bpm): mean (sd)	2	72.3 (11.9)	72.5 (11.9)	71.9 (12.1)	0.84^a^
Negative illness perception (IPQ) (0–10): mean (sd)	0	5.0 (1.4)	5.2 (1.4)	4.8 (1.2)	0.32^a^
Depression overall (BDI II) (0–100): median (IQR)	1	80.0 (60.0;94.0)	90.0 (60.0;98.5)	70.0 (60.0;90.0)	0.17^d^
Depression affective (BDI II) (0–100): median (IQR)	1	85.0 (70.0;98.0)	90.0 (60.0;99.0)	80.0 (70.0;90.0)	0.18^d^
Depression cognitive (BDI II) (0–100): median (IQR)	1	70.0 (50.0;90.0)	80.0 (50.0;95.0)	55.0 (50.0;85.0)	0.08^d^
Anxiety state (STAI-Y1) (0–100): median (IQR)	1	45.0 (39.0;52.0)	47.0 (37.5;53.5)	44.0 (40.5;47.3)	0.52^d^
Anxiety trait (STAI-Y2) (0–100): median (IQR)	1	45.0 (40.0;57.0)	46.0 (40.5;58.0)	43.5 (39.8;53.5)	0.62^d^
Sexual problems (SAS) (0–18): mean (sd)	5	6.7 (4.1)	7.2 (4.4)	6.0 (3.7)	0.30^a^
Need for sexual counselling (NSCS-CHF) (1–4): mean (sd)	10	2.4 (0.8)	2.4 (0.8)	2.3 (0.8)	0.77^a^

BMI, body mass index; NYHA, New York Heart Association; CHF, congestive heart failure; LVEF, left ventricular ejection fraction; IQR, inter-quartile range;

COPD, chronic obstructive pulmonary disease; CVA, cerebrovascular accident; TIA, transient transient ischemic attack; SCHFI, Self-Care of Heart Failure Index;

HRQoL, health-related quality of life; MLHFQ, Minnesota Living with Heart Failure Questionnaire; 6MWT, 6-min walking test; bpm, beats per minute.

^a^Result from independent samples Student’s t-test.

^b^Result from Chi² test.

^c^Result from Fisher’s Exact test.

^d^Result from Mann–Whitney *U* test.

The drop-out analysis comparing the 56 patients participating throughout the intervention period with the nine patients who were randomized but dropped out before or during the intervention period (see Fig. 1) showed no significant differences in any of the descriptive or outcome variables at T1 (*p* > 0.05; results not shown). Comparison at T1 between both countries showed a more adverse clinical profile in the Belgian (N = 34) vs. Italian (N = 22) sample, with a lower mean LVEF (30.2%, sd 6.4 vs. 34.8%, sd 5.2; *p* < 0.05), a higher median predicted 1-year mortality risk according to MAGGIC (28.1, IQR 14.6–39.7 vs. 9.3, IQR 6.3–14.7; *p* < 0.001), and higher mean values for impaired total HRQoL on the MLHFQ (36.0, sd 21.5 vs. 23.9, sd 13.8; *p* < 0.05) and impaired physical HRQoL on the MLHFQ (17.3, sd 10.7 vs. 9.6, sd 7.9; *p* < 0.01). On the other hand, Belgian patients scored significantly better on mean self-care maintenance score on the SCHFI at T1 (63.3, sd 13.9 vs. 54.1, sd 17.0; *p* < 0.05). No significant differences in socio-demographic characteristics, exercise capacity, or mental health at T1 were observed between the Belgian and Italian patient group (results not shown).

Intervention effects are reported in Table 2, showing the average change in outcome variables from T1 to T2. Self-care maintenance (SCHFI) significantly improved in the intervention group only, but the group difference was not significant. Self-care confidence on the SCHFI was not affected by the intervention. No intervention effects were observed for HRQoL (MLHFQ), exercise capacity (6MWT) or illness perception (IPQ). All mental health outcomes—i.e. depression (BDI II) and its sub-scales and both anxiety dimensions (STAI-Y1 and STAI-Y2)—improved significantly in the intervention group and showed significant group differences. The intervention group also experienced a significant reduction in sexual problems (SAS) from T1 to T2, while the need for sexual counselling (NSCS-CHF) significantly decreased in the control group. The additionally available clinical data showed a significant increase in LVEF, while the predicted one-year mortality risk scores significantly decreased in the intervention group. Further analysis showed no significant differences in descriptive characteristics or clinical profile between the groups with available vs. missing data (results not shown).

**Table 2:** Change in outcome variables from T1 to T2 in intervention and control group.

Outcome variables	Intervention group (N = 34)			Control group (N = 22)			*p-value g*roup difference
	Valid N	Change T2–T1^a^	*p-value*	Valid N	Change T2–T1^a^	*p-value*	
Self-care maintenance (SCHFI) (0–100): mean (sd)	**34**	**6.5 (15.0)**	** < 0.05**^**b**^	22	3.5 (15.8)	0.31^b^	0.48^d^
Self-care confidence (SCHFI) (0–100): mean (sd)	26	− 3.6 (14.3)	0.21^b^	19	− 7.9 (23.3)	0.16^b^	0.45^d^
Impaired HRQoL total (MLHFQ) (0–105): mean (sd)	34	− 1.0 (14.4)	0.70^b^	22	1.7 (13.8)	0.58^b^	0.50^d^
Impaired HRQoL physical (MLHFQ) (0–40): mean (sd)	34	− 0.4 (7.2)	0.74^b^	22	1.2 (5.9)	0.36^b^	0.39^d^
Impaired HRQoL emotional (MLHFQ) (0–25): mean (sd)	34	− 0.4 (5.4)	0.71^b^	22	0.2 (3.9)	0.83^b^	0.69^d^
Distance 6MWT (meters): mean (sd)	30	0.9 (88.8)	0.96^b^	17	4.8 (39.4)	0.62^b^	0.84^d^
Resting heart rate before 6MWT (bpm): mean (sd)	31	1.0 (11.1)	0.61^b^	17	0.4 (16.2)	0.92^b^	0.88^d^
Negative illness perception (IPQ) (0–10): mean (sd)	33	− 0.2 (1.2)	0.40^b^	22	− 0.03 (1.0)	0.90^b^	0.63^d^
Depression overall (BDI II) (0–100): median (IQR)	**33**	− **14.0 (**− **20.0;**− **7.0)**	** < 0.001**^**c**^	22	1.0 (− 9.3;10.0)	0.23^c^	** < 0.001**^**e**^
Depression affective (BDI II) (0–100): median (IQR)	**33**	− **10.0 (**− **19.5;**− **3.0)**	** < 0.001**^**c**^	22	0.0 (− 5.8;8.5)	0.52^c^	** < 0.001**^**e**^
Depression cognitive (BDI II) (0–100): median (IQR)	**33**	− **14.0 (**− **20.0;0.0)**	** < 0.001**^**c**^	**22**	**0.0 (0.0;20.0)**	** < 0.05**^**c**^	** < 0.001**^**e**^
Anxiety state (STAI-Y1) (0–100): median (IQR)	**32**	− **4.5 (**− **11.0;**− **0.5)**	** < 0.001**^**c**^	22	1.0 (− 2.0;2.3)	0.46^c^	** < 0.001**^**e**^
Anxiety trait (STAI-Y2) (0–100): median (IQR)	**33**	− **7.0 (**− **11.0;0.0)**	** < 0.001**^**c**^	22	0.0 (− 5.0;7.0)	0.66^c^	** < 0.01**^**e**^
Sexual problems (SAS) (0–18): mean (sd)	**29**	− **1.9 (3.7)**	** < 0.05**^**b**^	18	− 0.8 (3.2)	0.28^b^	0.32^d^
Need for sexual counselling (NSCS-CHF) (1–4):mean (sd)	27	0.1 (0.8)	0.32^b^	**16**	− **0.4 (0.7)**	** < 0.05**^**b**^	** < 0.05**^**d**^
LVEF (%):mean (sd)	**21**	**3.4 (7.0)**	** < 0.05**^**b**^	16	− 0.4 (5.8)	0.78^b^	0.08^d^
MAGGIC (% 1-year mortality risk): median (IQR)	**29**	− **1.2 (**− **8.1;0.6)**	** < 0.01**^**c**^	15	− 0.4 (− 8.2;1.0)	0.20^c^	0.52^e^
3C-HF (% 1-year mortality risk): median (IQR)	**30**	− **0.5 (**− **5.3;0.3)**	** < 0.05**^**c**^	21	0.0 (− 7.0;1.5)	0.08^c^	0.75^e^

SCHFI, Self-Care of Heart Failure Index; HRQoL, health-related quality of life; MLHFQ, Minnesota Living with Heart Failure Questionnaire; 6MWT, 6-min walking test;

bpm, beats per minute; IQR, inter-quartile range; LVEF, left ventricular ejection fraction.

Significant results are shown in bold.

^a^Positive value indicates increase from T1 to T2, while negative value indicates decrease from T1 to T2.

^b^Result from paired-samples Student’s t-test.

^c^Result from Wilcoxon signed-rank test.

^d^Result from independent samples Student’s t-test.

^e^Result from Mann Whitney *U* test.

## Discussion

In the past years the use of tele-monitoring systems in cardiac patients has increased tremendously^25^. However, their effectiveness in managing CHF patients remains controversial, and both positive results^26^ as lacking effects^7,9,10^ on mortality and hospitalisation have been reported. Real-time transmission of patient data enables remote follow-up, but involves substantial interaction with healthcare providers. These systems do not focus on empowering patients to properly manage their disease. There is high need for systems that focus on self-care and enhance individual adherence of patients to their complicated treatment plans and lifestyle advice. Patients who are actively involved in their own care, and who adhere to the medication and lifestyle regimen, are known to have better prognosis^6^. So in addition to pure tele-monitoring, there is a need for mHealth systems that interpret data generated in home environments directly for the benefit of the patients, but the implementation of DSSs in the context of patient support in home environments is still limited^27^. HeartMan was designed as an advanced disease management support system for stable CHF patients to use in their home environment^11^. End users were involved in all development stages using a human-centred design process. Its major strengths are the personalisation to the individual patient’s clinical and psychological profile, and its advanced functionality by integrating several relevant intervention modalities relating to physical exercise, nutrition, medication intake, mental support and disease education.

The results showed that HeartMan was effective in improving mental health in CHF patients. The psychological support module in the HeartMan system was quite advanced, including personalized messages based on cognitive behavioral therapy (CBT) to dissolve patients’ cognitive dissonance, in addition to a weekly plan of mindfulness games and exercises for relaxation and general psychological wellbeing^11^. The findings suggest that psychological support had an important impact on patients, relieving their somatic-affective and cognitive symptoms of depression (BDI II), as well as their state and trait signs of anxiety (STAI-Y). This is in line with the growing evidence showing the ability of mindfulness to improve psychological well-being in chronic disease overall^28^, and specifically in CHF^29^. Although the available evidence from RCT studies is limited and somewhat mixed, several reviews show that CBT strategies can have beneficial effects on mental health and symptom burden in CHF populations^30–32^.

Results of this trial further suggest that using HeartMan improved self-care behaviour. The self-care maintenance score of the SCHFI, indicating overall quality of disease management on a scale from 0 to 100, improved significantly in the intervention group with 6.5 points, which is a little below the threshold of half a standard deviation for a clinically relevant change^16^. In line with documented poor levels of adherence and self-management in this patient group^33^, the overall level at T1 was below the 70 point cut-off for adequate self-care in both intervention and control group. It approached the threshold of 70 in the intervention group at T2, which is suggested to be the minimal effectiveness cut-point for improving health outcomes^16^. It should be noted though that no significant group difference was observed for this outcome, so this result should be interpreted with caution.

The mobile application provided access to a depository which users could consult on a voluntary basis to look up educational information about CHF and its treatment, as well as expert knowledge about how the disease impacts on sexual activity and dysfunction^11^. Problems with sexual functioning and activity are very common in CHF, while satisfaction with one’s sexual activity is considered important by most patients^34,35^. We observed a reduction in experienced sexual problems in the intervention group in addition to a significant difference in need for sexual counselling which decreased in the control group only. These findings suggest that applying mHealth for sexual counselling—combined with the observed improvement in self-care—might evoke reflection upon the topic and lower the experience of sexual problems. It should be noted though that about 1/5 had incomplete data on these questionnaires, presumably because some felt uncomfortable answering these questions. Therefore, we need to interpret these results with caution since a selection bias is likely, i.e. those who answered the questionnaires are probably those with better outcomes.

Null findings were observed on the remaining primary and secondary endpoints, i.e. HRQoL, exercise capacity and illness perception. Although most mHealth interventions focus on hard endpoints rather than PROs, the large-scale BEAT-HF trial showed an improvement in HRQoL, while no effect on hospital readmission was observed^10^. We used the same instrument to measure HRQoL, i.e. the MLHFQ, but a much smaller sample was involved. The null finding for the emotional dimension of HRQoL is in contrast with the observed intervention effect on depression and anxiety. These conflicting results are against expectations since depressive symptoms have been identified as consistent predictors of HRQoL in CHF patients^36^. A plausible explanation lies in the different characteristics of the instruments used. The MLHFQ includes five items to cover the emotional dimension of impaired HRQoL, asking rather explicitly about a patient’s overall emotional state^17^. As such it is less specific and significantly affected by the patient’s momentary perception^37^, compared to the BDI II and STAI-Y instruments which are more accurate (incorporating 21 and 40 items, respectively) and sensitive to evaluate psychological predictors of depression or anxiety^19,20^.

The comprehensive physical exercise program in the HeartMan DSS was adapted to each patient's physical capacity and psychological profile, and provided a gradually increasing weekly set of endurance and resistance exercises^11^. No effect on exercise capacity was observed, neither on distance walked in the 6MWT nor on resting heart rate. Our results suggest that while the HeartMan system empowered patients to improve their self-care behavior and advanced their mental health, the system did not sufficiently succeed in making them adhere to the advanced exercise program. Although there is convincing evidence about the benefits of exercise in CHF, the uptake of exercise is known to be problematic^5,33^.

Wearables and mHealth applications providing individualized follow-up to patients in their maintenance phase of cardiac rehabilitation have been shown effective for improving cardiorespiratory fitness and exercise capacity^38,39^. However, in a group of stable CHF patients with large variety in exercise habits at baseline, the initiation of a detailed exercise program through a mobile application, even if it is individualized and gradually progressive, is much more challenging. The effects may thus be less pronounced and remain undetected in this small proof-of-concept trial, especially since 6 patients (3 in each group) with baseline information were not able to perform the 6MWT at T2. Regarding the use of a 6MWT, further research with cardiopulmonary exercise testing as the state-of-the-art method for measuring exercise capacity will be required.

Contrary to what we hypothesized, the HeartMan intervention did not affect patients’ illness perception (IPQ) nor their level of self-care confidence (SCHFI). The disease education module provided in the depository of the mobile application was meant to improve patients’ comprehension of the disease and to boost their confidence to take actions and self-manage their CHF. Negative illness perceptions are known to be associated with worse clinical outcomes, underlining the importance of changing these representations as a treatment goal^40^. Again it is possible that the sample was too small or the intervention period too short to induce significant effects on these outcome measures.

The main limitation of this proof-of-concept trial is the small sample size resulting in preliminary findings. A priori sample size calculations indicated a target number of 90 patients^11^. While a total of 79 patients signed informed consent, only 56 were included in the final effect evaluation. No major drop-out effects were observed when comparing the final sample with those who dropped out after randomisation, although the small and unbalanced numbers in this analysis make reliable conclusions difficult. Strict inclusion and exclusion criteria were adopted, and especially the restriction to patients with reduced LVEF ≤ 40% critically limited the number of eligible patients. Another major bottleneck was the common reluctance of patients to participate in a randomized study in general, or to enter an intervention program requiring intensive use of an mHealth system. Therefore, the number of eligible and consenting patients was much lower than anticipated in both countries, and the restrictions imposed by the funding agency did not allow any extension of the recruitment period. A second limitation is that inevitably a number of technical difficulties or flaws arose in this proof-of-concept trial where the system was evaluated in a real patient setting for the first time. A helpdesk was operational for addressing technical issues and problems with using the system, but nonetheless this likely affected some patients’ adherence to the system. As a result, some of the truly potential intervention effects may have been underestimated. Because of the multi-modal nature of the system only an overall effect evaluation was possible, disregarding the effects of the individual components. Also, there is a risk of multiple testing problem as we targeted multiple endpoints in relation to the multi-disciplinary system. The system was tested in patients from two countries, increasing the generalizability of findings. A number of characteristics differed between the Belgian and Italian samples at T1, but the low number of participants does not allow analysing stratified intervention effects. Overall, the HeartMan patient population was relatively young, including mostly men with mild symptoms.

In conclusion, the Heartman system significantly improved mental health and a trend for improved self-care behaviour was observed, outcomes which are known to not only reinforce one another but also predict hard clinical endpoints in CHF^6,33^. In contrast, no effects on HRQoL, exercise capacity and illness perception were detected. In addition to the pre-defined endpoints indicated in the study protocol, additional electronic health record data enabled to verify potential effects of the system on clinical outcomes. Follow-up data on LVEF and predicted one-year mortality risk were available for more than two thirds of the sample and thus provided preliminary findings about the clinical effectiveness. Patients in the intervention group significantly improved their LVEF and the difference with the control group was borderline significant. A new assessment of LVEF at follow-up was not available in 38% of the intervention and 27% of the control group, and although no systematic bias seemed present, caution is required in the interpretation of this finding. Intervention patients significantly reduced their predicted one-year mortality risk according to the MAGGIC^23^ and 3C-HF^24^ scoring systems, although similar trends were observed in the control group. These preliminary findings need to be investigated more in depth. Future validation studies are needed to test the overall effectiveness of the HeartMan system in a wider context, i.e. in larger samples of CHF patients using a less restricted participant profile and applying a longer intervention period.

## Data Availability

The datasets generated during and analysed during the current study are available from the corresponding author on reasonable request.

## References

[1] Ponikowski P (2014). Heart failure: preventing disease and death worldwide. ESC Heart Failure.

[2] Ponikowski P (2016). ESC guidelines for the diagnosis and treatment of acute and chronic heart failure: the task force for the diagnosis and treatment of acute and chronic heart failure of the European Society of Cardiology (ESC). Developed with the special contribution of the Heart Failure Association (HFA) of the ESC. Eur. J. Heart Failure.

[3] Roger VL (2013). Epidemiology of heart failure. Circ. Res..

[4] Zannad F (2013). Clinical outcome endpoints in heart failure trials: a European Society of Cardiology Heart Failure Association consensus document. Eur. J. Heart Fail.

[5] Piepoli MF (2011). Exercise training in heart failure: from theory to practice. A consensus document of the Heart Failure Association and the European Association for Cardiovascular Prevention and Rehabilitation. Eur. J. Heart Fail.

[6] Lainscak M (2011). Self-care management of heart failure: practical recommendations from the patient care committee of the heart failure association of the European society of cardiology. Eur. J. Heart Fail.

[7] Chaudhry SI (2010). Telemonitoring in patients with heart failure. N. Engl. J. Med..

[8] Inglis, S.C., Clark, R.A., Dierckx, R., Prieto‐Merino, D., & Cleland, J.G. Structured telephone support or non‐invasive telemonitoring for patients with heart failure. *Cochrane Database Syst. Rev.* (2015). 10.1002/14651858.CD007228.pub3 PMC8482064 26517969

[9] Koehler F (2011). Impact of remote telemedical management on mortality and hospitalizations in ambulatory patients with chronic heart failure: the telemedical interventional monitoring in heart failure study. Circulation.

[10] Ong MK (2016). Effectiveness of remote patient monitoring after discharge of hospitalized patients with heart failure: the better effectiveness after transition–heart failure (BEAT-HF) randomized clinical trial. JAMA Int. Med..

[11] Baert A (2018). A personal decision support system for heart failure management (heartman): study protocol of the heartman randomized controlled trial. BMC Cardiovasc. Disord..

[12] Anker SD (2014). The importance of patient-reported outcomes: a call for their comprehensive integration in cardiovascular clinical trials. Eur. Heart J..

[13] Chamberlain AM (2014). Self-rated health predicts healthcare utilization in heart failure. J. Am. Heart Assoc.

[14] Monzo L (2020). Association of patient-reported outcomes and heart rate trends in heart failure: a report from the Chiron project. Sci. Rep..

[15] Mlakar M (2018). Mining telemonitored physiological data and patient-reported outcomes of congestive heart failure patients. PLoS ONE.

[16] Riegel B, Lee CS, Dickson VV, Carlson B (2009). An update on the self-care of heart failure index. J. Cardiovasc. Nurs..

[17] Rector T, Francis G, Cohn J (1987). Patients self-assessment of their congestive heart failure Part 1: patient perceived dysfunction and its poor correlation with maximal exercise tests. Heart Failure.

[18] Broadbent E, Petrie KJ, Main J, Weinman J (2006). The brief illness perception questionnaire. J. Psychosom. Res..

[19] Beck AT, Steer RA, Brown GK (1996). Beck depression inventory-II. San Antonio.

[20] Spielberger, C.D. State-trait anxiety inventory for adults. (1983).

[21] Derogatis LR (1986). The psychosocial adjustment to illness scale (PAIS). J. Psychosom. Res..

[22] Driel AG, de Hosson MJJ, Gamel C (2014). Sexuality of patients with chronic heart failure and their spouses and the need for information regarding sexuality. Eur. J. Cardiovascular Nursing.

[23] Pocock SJ (2013). Predicting survival in heart failure: a risk score based on 39 372 patients from 30 studies. Eur. Heart J..

[24] Senni M (2013). Predicting heart failure outcome from cardiac and comorbid conditions: the 3C-HF score. Int. J. Cardiol..

[25] Monzo, L., Schiariti, M. & Puddu, P.E. Wireless Telecardiology. in *Health Monitoring Systems: An Enabling Technology for Patient Care* (eds. Gupta, R. & Biswas, D.) (CRC Press, 2019).

[26] Lin M-H (2017). Clinical effectiveness of telemedicine for chronic heart failure: a systematic review and meta-analysis. J. Investig. Med..

[27] Marschollek M (2012). Decision support at home (DS@ HOME)–system architectures and requirements. BMC Med. Inform. Decis. Mak..

[28] Greeson JM, Chin GR (2019). Mindfulness and physical disease: a concise review. Current Opin. Psychol..

[29] Norman J, Fu M, Ekman I, Björck L, Falk K (2018). Effects of a mindfulness-based intervention on symptoms and signs in chronic heart failure: A feasibility study. Eur. J. Cardiovasc. Nurs..

[30] Jeyanantham K, Kotecha D, Thanki D, Dekker R, Lane DA (2017). Effects of cognitive behavioural therapy for depression in heart failure patients: a systematic review and meta-analysis. Heart Fail. Rev..

[31] Kwekkeboom KL, Bratzke LC (2016). A systematic review of relaxation, meditation, and guided imagery strategies for symptom management in heart failure. J. Cardiovasc. Nurs..

[32] Lundgren J, Andersson G, Johansson P (2015). Can cognitive behaviour therapy be beneficial for heart failure patients?. Curr. Heart Fail. Rep..

[33] Corotto PS, McCarey MM, Adams S, Khazanie P, Whellan DJ (2013). Heart failure patient adherence: epidemiology, cause, and treatment. Heart Fail. Clin.

[34] Baert A (2019). Sexual activity in heart failure patients: information needs and association with health-related quality of life. Int. J. Environ. Res. Public Health.

[35] Schwarz E (2008). The prevalence and clinical relevance of sexual dysfunction in women and men with chronic heart failure. Int. J. Impot. Res..

[36] Baert A (2018). Factors associated with health-related quality of life in stable ambulatory congestive heart failure patients: Systematic review. Eur. J. Prevent. Cardiol..

[37] Purves D (2001). Physiological changes associated with emotion.

[38] Hannan AL (2019). Impact of wearable physical activity monitoring devices with exercise prescription or advice in the maintenance phase of cardiac rehabilitation: systematic review and meta-analysis. BMC Sports Science, Medicine and Rehabilitation.

[39] Lunde, P.*, et al.* Long-term follow-up with a smartphone application improves exercise capacity post cardiac rehabilitation: a randomized controlled trial. *Eur. J. Prevent. Cardiol.* 2047487320905717 (2020). 10.1177/2047487320905717 PMC7564298 32106713

[40] Hagger MS, Orbell S (2003). A meta-analytic review of the common-sense model of illness representations. Psychol. Health.

